# Enrichment of bacterial DNA for the diagnosis of blood stream infections

**DOI:** 10.1186/s12879-016-1568-1

**Published:** 2016-05-31

**Authors:** Ngo Tat Trung, Tran Thi Thu Hien, Tran Thi Thanh Huyen, Dao Thanh Quyen, Trinh Van Son, Phan Quoc Hoan, Nguyen Thi Kim Phuong, Tran Thi Lien, Mai Thanh Binh, Hoang Van Tong, Christian G. Meyer, Thirumalaisamy P. Velavan, Le Huu Song

**Affiliations:** Department of Molecular Biology, 108 Military Central Hospital, Hanoi, Vietnam; Institute of Clinical Infectious Diseases, 108 Military Central Hospital, Hanoi, Vietnam; Department of Clinical Microbiology, 108 Military Central Hospital, Hanoi, Vietnam; Faculty of Infectious Diseases, Hai Phong Medical University, Hai Phong, Vietnam; Department of Gastroenterology, 108 Military Central Hospital, Hanoi, Vietnam; Institute of Tropical Medicine, University of Tübingen, Tübingen, Germany; Vietnamese - German Centre for Medical Research (VG-CARE), Hanoi, Vietnam

**Keywords:** Sepsis, Bloodstream infection, Human DNA removal, Molecular diagnosis, Blood culture

## Abstract

**Background:**

Blood cultures are commonly employed to identify bacterial pathogens causing sepsis. PCR assays to diagnose septicemia require extraction of bacterial DNA from blood samples and thus, delay the initiation of appropriate antimicrobial treatment. The presence of abundant human DNA may hamper the sensitivity of PCR in the detection of bacteria.

**Methods:**

We used serial dilutions of *E. Coli* spiked pseudo-blood-sepsis samples to develop a simple method that combines the use of a polar detergent solvent and adjustment of the basic pH to remove human DNA. A 16S rRNA gene-based screening algorithm was established to differentiate Gram-positive and Gram-negative groups of bacteria and the family of Enterobacteriaceae. A stringent validation with appropriate controls was implemented. The method of human DNA removal was then applied on 194 sepsis blood samples and 44 cerebrospinal fluid (CSF) samples by real-time PCR.

**Results:**

This uncomplicated and straightforward approach allows to remove up to 98 % of human DNA from peripheral blood of septic patients. The inhibitory effect of human DNA is efficiently prevented and the detection limit of real-time PCR is increased to 10 *E. Coli* CFUs/ml. This sensitivity is 10 times higher compared to conventional real-time PCR assays. The classical blood culture detected 58/194 (30 %) of sepsis and 9/44 (21 %) of CSF samples. Out of the 194 blood samples tested, the conventional real-time PCR targeting 13 common sepsis causing pathogens correctly detected the bacterial DNA in 16/194 (8 %) only and 14/44 (32 %) in cerebrospinal fluid samples. Our newly established approach was able to provide correct diagnoses in 78 (40 %) of the 194 blood samples and in 14 (32 %) of the CSF samples. The combination of both blood cultures and our technique raised the rate of sepsis diagnoses to 112/194 (58 %). Of the total group tested positive, 46 (24 %) cases showed overlap with the classical methodology.

**Conclusion:**

We report a simple optimized in-house protocol for removal of human DNA from blood sepsis samples as a pre-analytical tool to prepare DNA for subsequent PCR assays. With the detection increase of our in-house DNA removal approach, subsequent PCR assays can reach detection limits of 10 *E. coli* CFUs/ml and significantly improve the diagnostic rate in blood sepsis cases.

**Electronic supplementary material:**

The online version of this article (doi:10.1186/s12879-016-1568-1) contains supplementary material, which is available to authorized users.

## Background

Even after the development of new diagnostic and therapeutic techniques, the physiopathology of sepsis is still poorly understood; incidences and mortality are steadily increasing. Delayed diagnosis usually results in multi-organ failure and with frequent fatal outcomes [1]. Timely and appropriate diagnoses of bloodstream infections (BSI) have a significant and measurable impact on patient care and infection control in resource-limited settings [2]. Although the classical blood culture is widely accepted as a gold standard it still has various drawbacks. For instance, blood cultures are time-consuming and can only detect bacteria growing on suitable media used. A minimum volume of 30 ml of blood is required for each aerobic and anaerobic bacterial cultures [3]; such amounts are challenges particularly for old and neonatal patients. A rapid diagnostic method applicable under clinical conditions and complementing classical blood cultures is therefore essential.

The polymerase chain reaction (PCR) has provided new possibilities for rapid detection of bacterial pathogens in patients with sepsis [2]. In addition, the multiple-priming format (multiplex) PCR allows rapid detection of various microbial pathogens in a shorter period of time. Although many advancements in molecular diagnostics of bacterial pathogens have been achieved [4, 5], PCR based diagnoses of BSI are still difficult. A most critical factor is that PCR assays may fail to amplify sparse copies of pathogens in the abundance of human DNA. In addition, PCR-inhibitory compounds in human blood can considerably reduce the sensitivity of an assay. For instance, a typical BSI case has a bacterial density of 1-1000 CFU/ml in their peripheral blood [6]. An optimized PCR reaction can only amplify the 16S rRNA target if the bacterial load exceeds 1000 CFU/ml. Thus, PCR assays may be ineffectual in replicating blood culture results [7, 8]. The diversity of bacterial strains further complicates optimized conditions for multiplex PCR reactions.

During evolution, the fusion of two prokaryotic genomes (e.g., Archae and Proteobacteria) has resulted in the formation of a eukaryotic genome with sufficient horizontal gene transfer [9, 10]. This indicates that the possibility of the presence of homologous and/or conserved DNA sequences between human and bacterial genomes exists, possibly causing primer mispairing and consequently leading to false positive results. Commercial molecular diagnostic tests such as the SepsiTest (Molzym), and VYOO (SIRS Lab) apply various approaches to deplete human genomic DNA prior to the molecular diagnosis [7, 11–14]. In these molecular assays, either DNAses cleaving human DNA or methylated chromatography columns are used to remove human DNA. Larger volumes of human blood are recommended in these assays. Since such kits are costly, low-income countries can frequently not afford them.

We developed a simple in-house method that combines the use of a polar detergent solvent and adjustment of basic pH to remove human DNA. We also utilized a 16S rRNA based real-time PCR screening algorithm to differentiate Gram-positive and Gram-negative bacteria as well as the family of Enterobacteriaceae. Our newly developed procedure was validated in 194 peripheral blood samples obtained from septic patients with likely abundant human DNA and 44 samples of cerebrospinal fluid (CSF) with sparse human DNA. The diagnostic performance of i) the classical blood culture approach (arm-1), ii) conventional real-time PCR screening using total DNA extracted from patients samples (arm-2), and iii) our real-time PCR screening approach using templates depleted of human DNA (arm-3) were compared.

## Methods

### Ethics statement

The study was submitted for regulatory approval to the institutional review board of the 108 hospital and was approved. Following submission, the Ethical Committee of the 108 Military Central Hospital, Hanoi has provided consent and ethical approval for the study was duly obtained. Informed written consent was obtained from all study patients and/or from their parents if the study participant was under 18 years of age.

### Dilution series formulation

The *E. coli* DH5 alpha strain (Invitrogen, Singapore) was inoculated into liquid LB medium for proliferation until reaching the log phase. A dilution series from 10000 CFU/mL to 1 CFU/mL until eventual negativity was done to spike the bacteria into 5 mL of human blood. The spiked bacteria in blood were then used for the procedure of human DNA depletion and subsequent isolation of bacterial DNA. DNA extracted from single colonies of *Klebsiella pneumoniae, Proteus mirabilis, Neisseria meningitidis, Salmonella spp., Pseudomonas aeruginosa, Acinetobacter baumannii, Staphylococcus aureus, S. epidermidis, Streptococcus pneumoniae, S. suis* and *Enterococcus spp.* were reconstituted in 200 μl of 25 mM Tris-EDTA at pH 8 containing 100 ng/μl human DNA extracted from full blood; 5 μl of these solutions were used as positive controls for all PCR or real-time PCR reactions.

### Depletion of human DNA and bacterial DNA isolation

Several detergents including non-ionic (NP-40, Triton-X 100), ionic (CTAB, SDS) and zwitterionic (CHAPS) detergents were screened. Only Triton-X 100 was able to lyse mammalian cell membranes, but not those of *E. coli*. Triton-X 100 was, therefore, considered for further analyses. Different conditions such as variation in pH, time, and Na_2_CO_2_ concentrations were also optimized (Additional file 1: Table S1). Mammalian cell lysis buffers MCLB-1 was used to treat the pseudo-sepsis samples by mixing volumes of 1.2 ml blood from healthy donors (spiked with 100,000 *E. coli* cells) with 1.2 ml solvent. After solvent treatment, the suspensions were centrifuged to collect bacterial pellets for subsequent NaOH/SDS total DNA extraction. Residuals of human DNA and intactness of spiked *E. coli* genome were examined and quantified by RT-PCR (Additional file 1: Figures S1 and S2).

Overnight-grown *E. coli* were spiked in peripheral venous blood samples obtained from healthy donors to establish dilution serials of 1000 CFU/ml, 100 CFU/ml, 10 CFU/ml, 1 CFU/ml and 0 CFU/ml (negative control). The dilution serials were separated into two groups. The first group was subjected to human DNA removal by mixing thoroughly with an equal volume of mammalian cell lysis buffer (MCLB-1, containing 2 M Na_2_CO_2_ pH 9.8, 1 % Triton-X100) for three minutes at ambient temperature to allow for complete fragmentation of human chromatin into DNA fragments. After the incubation step an equal volume of neutralization buffer (1 M Tris–HCl, pH 4.5) was added in order to prevent further cell lysis. The samples were then centrifuged at 5000 g for 5 min. The supernatants were discarded and bacterial pellets were resuscitated in 200 μl of 1x phosphate buffered saline (PBS) and used for isolation of bacterial DNA by a conventional method using the NaOH/SDS approach [15]. The second group of the dilution serials also underwent conventional DNA extraction using the NaOH/SDS approach [15].

The integrity of spiked *E. coli* genomic DNA and the presence of residual human DNA was measured by *E. coli* specific primers/probe (Additional file 1: Tables S2 and S3) real-time PCR or SYBR green based real-time PCR using the beta globin-specific primers: BetaF: 5’-AGA AGA GCC AAG GAC AGG TAC G-3’, BetaR: 5’-TGC TAG TGA ACA CAG TTG TGT CAG A-3’.

### Reagent preparation under aseptic conditions

Primers and labeled probes (Integrated DNA Technologies, Singapore) used are listed in Additional file 1: Tables S2 and S3. All primers and probes were available in stocks of 100 pmol/μl in 25 mM Tris–HCl and treated with 1 U DNase/20 μl reaction volume for 15 min at 37 °C. Subsequently, the DNAse was inactivated by heating at 95 °C for 40 min. After inactivation, the stock was aliquoted into volumes of 10 μl and stored at −20 °C [16]. For decontamination of the PCR mastermix, 8-methoxypsoralen was dissolved in dimethylsulfoxide (DMSO) (Sigma, Germany). For decontamination of PCR water and the real-time PCR mastermix reagents, 25 μg/mL of 8-methoxypsoralen (Sigma, Germany) and 10 min exposure to 366 nm UV irradiation over a distance of 3 cm were applied. The MCLB-1 buffer was also decontaminated by UV irradiation at 280 nm over a distance shorter than 5 cm [16]. Working places and all accessories and tools in our laboratory are decontaminated weekly by spraying with DNA-ExitusPlus™ solution (AppliChem, Germany).

### Real-time PCR conditions

The real-time PCR assay mixtures consisted of 7.5 μl Taqman real-time PCR Master Mix (Qiagen, Hilden, Germany), 5 μl DNA template, 5 pmol of primers and 0.2 pmol of probe. The reaction was run in the Stratagene M3000p (Stratagene, USA) device with a preincubation step at 50 °C for 15 min, initial denaturation at 95 °C for 5 min, followed by 45 cycles of 95 °C for 15 s and 60 °C for 60 s. The first real-time PCR assay differentiates Gram-negative, Gram-positive and Enterobacteriacea bacteria. The second assay discriminates the distinct bacterial species using primers and Taqman probes specific for the 13 most common pathogens, namely *Staphylococus aureus, S. epidermidis, Streptococcus pneumoniae, S. suis, Enterococcus spp.* (Gram-positive bacteria) and *Escherichia coli, Klebsiella pneumoniae, Proteus mirabilis, Salmonella spp., Neisseria meningitidis, Pseudomonas aeruginos,* and *Acinetobacter baumannii* (Gram-negative bacteria).

### Sepsis patients and blood sampling

A total of 194 peripheral venous blood and 44 CSF from septic patients were available at the 108 Military Central Hospital, Hanoi, Vietnam. Samples were collected between February 2014 and September 2015. Diagnoses of sepsis were confirmed by physicians, based on a body temperature above 38.3 °C or below 36 °C, heart rate >90 beats per minute, respiratory rate higher than 20 breaths per minute, an assumed or confirmed infection with at least one of the following signs and symptoms: significantly decreased urine output, abrupt change in consciousness/mental status, decreased platelet count, difficulty of breathing, abnormal heart pumping function, or abdominal pain [17]. Blood and CSF samples from all patients were preserved in heparinized tubes. Prior to the analyses specimens were stored at 4 °C.

**Fig. 1:**
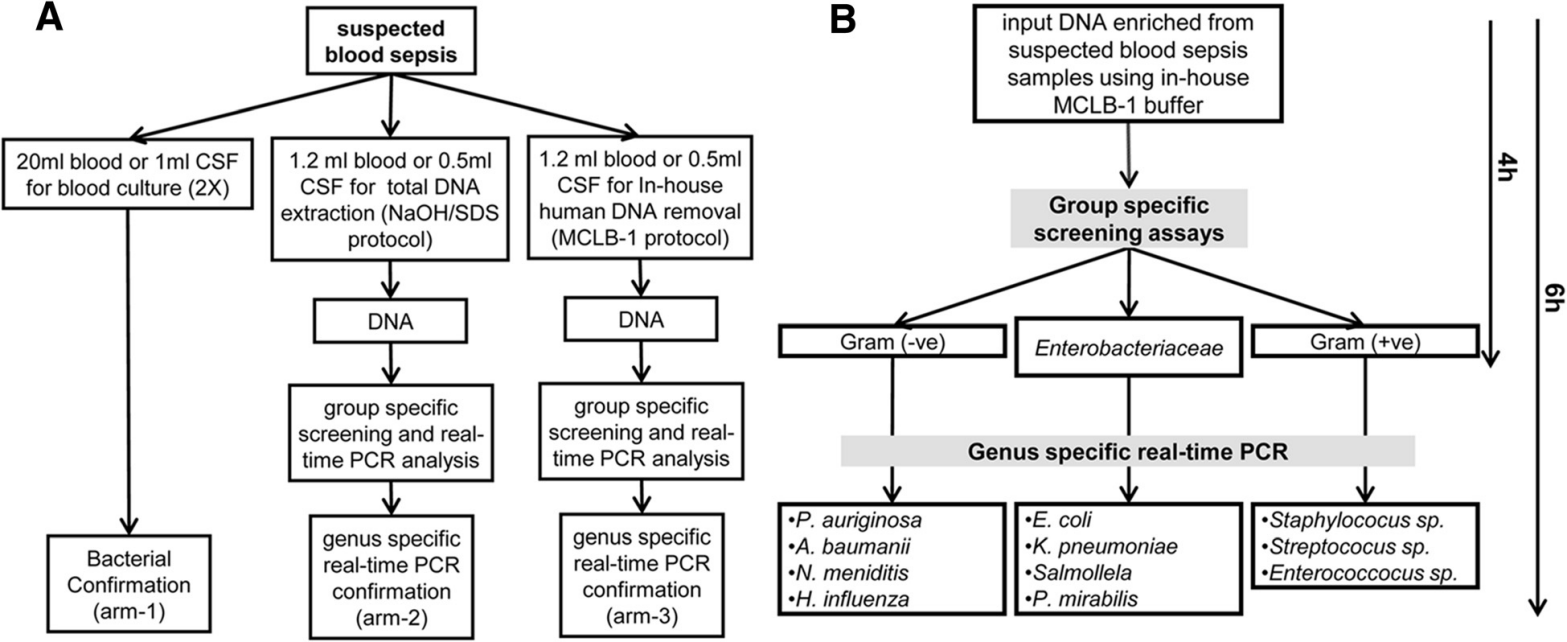
Work flow of the established protocol for human DNA removal. **a** Study flowchart to compare sepsis causing pathogen diagnoses by PCR and blood culture. **b** Group-specific screening by PCR reactions targeting bacterial 16SrRNA gene to differentiate Gram-positive, Gram-negative and Enterobacteriaceae groups. Samples positive in the screening assay were subjected to genus-specific real-time PCR reactions to detect 13 most common sepsis causative pathogens

In order to compare diagnostic techniques in the identification of pathogens causing sepsis, 10 ml of venous blood or 0.5 ml CSF were collected in duplicates from all patients and divided into equal portions for the detection of aerobic bacteria. The blood samples were subjected to automatic bacterial culture, and CSF was used for conventional bacterial cultures. In parallel, 2.4 ml blood or 1 ml of CSF were equally partitioned into one sample (1.2 ml blood or 0.5 ml CSF) that was used directly for total DNA extraction, and a second sample (1.2 ml blood or 0.5 ml CSF) which was used for MCLB-1 treatment to remove human DNA prior to extraction of bacterial DNA. DNA isolated from samples treated with MCLB-1 was used for screening to discriminate Gram-negative, Gram-positive and Enterobacteriacea bacteria. Samples positive in the screening step were then subjected to group-specific real-time PCR assays to identify and confirm infection by 13 common pathogens causing sepsis as mentioned above (Fig. 1a).

### Automated bacterial culture

Twenty ml blood were collected per patient and aliquoted in 10 ml tubes for culturing in the BD BACTEC™ 9120 blood culture system at 36 °C ± 0.5 for 48 h until growth in both tubes was observed (Becton Dickinson, New Jersey, USA). After 24 h of incubation, identification of bacterial species was performed using the VITEK® 2 automated system (BioMérieux, France).

#### Conventional bacterial culture

To establish diagnostic techniques for the examination of CSF samples, we followed protocols provided by the Manual of Clinical Microbiology [18] and the CDC Laboratory Methods for the Diagnosis of Meningitis [19]. Briefly, 600 μl aliquots of CSF from each patient was vortexed in 600 μl PBS. The resulting suspension was streaked onto four media, namely blood agar, MacConkey agar, chocolate agar and buffered charcoal-yeast extract agar (BCYE) and incubated under aerobic conditions at 37 °C for three days. Colonial growth was asserted and confirmed by microbiologists.

## Results

### Efficiency of human DNA removal

**Fig. 2:**
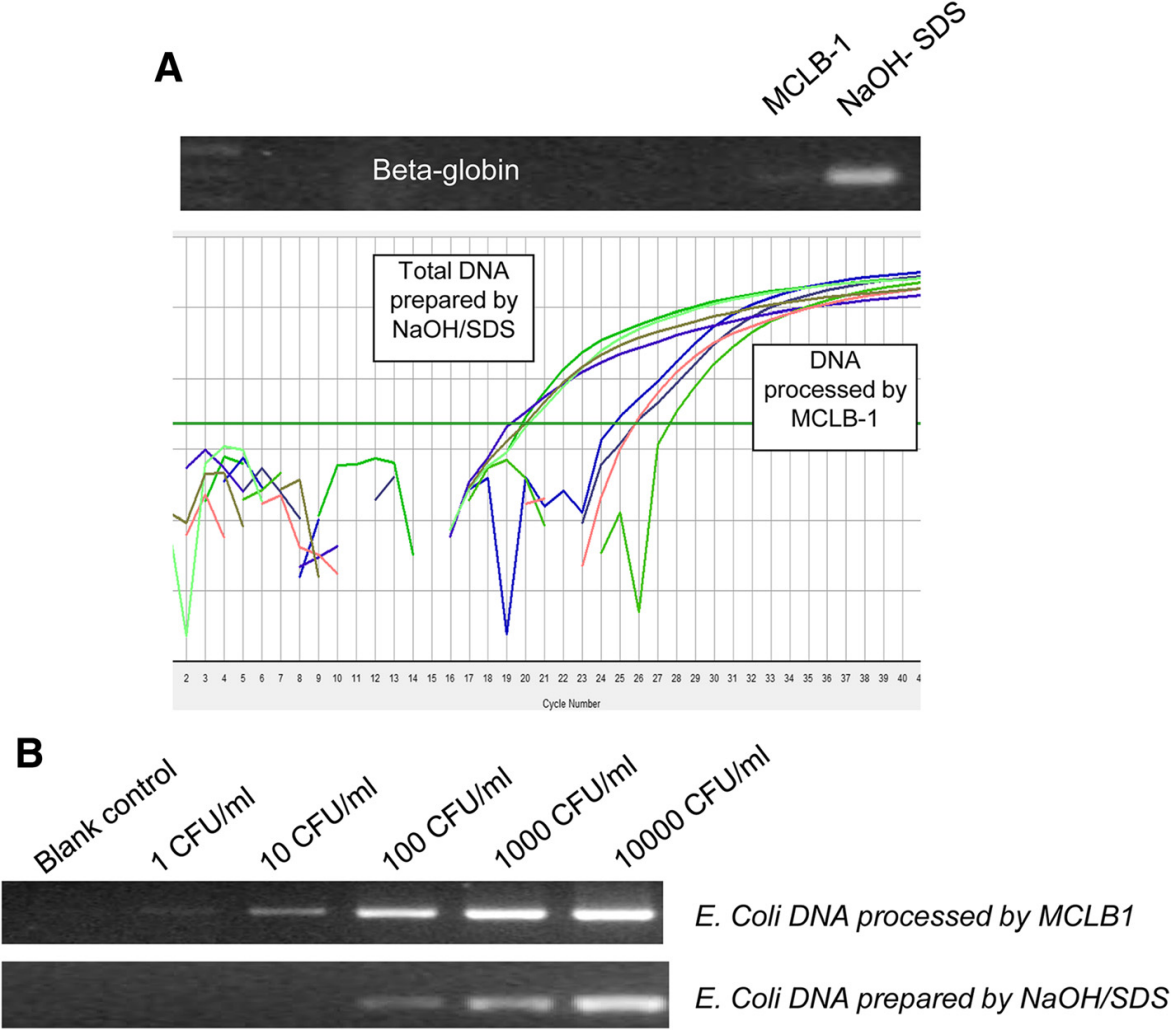
Efficiency of human DNA removal and sensitivity of PCR assay. **a** Residues of human DNA after MCLB-1 treatment were monitored via beta-globin derived amplification assays. Upper panel gel based PCR assay targeting the beta-globin gene; lower panel is SYBR green based real-time PCR to quantify residual beta-globin gene fragments. **b** Detection limits of spiked *E. coli* at various densities. At high density of spiked bacteria (1000 CFU/ml or 10000 CFU/ml), removal of human DNA does not provide a significant diagnostic signal, but at low concentration (100 CFU/ml or 10 CFU), removal of human DNA enhances detection limit of the diagnostics PCR to 10 CFU/ml

Utilization of mammalian cell lysis buffer (MCLB-1) resulted in removal of 98 % human DNA, obviously without affecting bacterial DNA (Fig. 2a). Our data reveal that, at a high density of spiked bacteria (>1000 CFU/ml), human DNA does not impede the downstream PCR. However, at lower bacterial loads (<100 CFU/ml) human DNA inhibits subsequent PCR reactions (Fig. 2b, Table 1, Additional file 1: Figure S3). Efficient removal of inhibitory effects caused by human DNA increased the detection limits of the PCR to 10 CFU/ml, a detection limit which conventional PCR assays could not reach (Fig. 2, Table 1). The discrimination of Gram-positive and Gram-negative bacteria as well as Enterobacteriacea requires four hours only, with two additional hours of the real-time PCR assay to identify bacterial species (Fig. 1b).

**Table 1:** Ct value of spiked *E. coli* dilution series upon in-house human DNA removal step

*E. coli* density (CFU/ml)	1000	100	10	1	0
MCLB1 processed DNA	29.2 ± 0.5	34 ± 0.5	37 ± 1	Undetectable^(a)^	Undetectable^(a)^
NaOH/SDS processed DNA	29.0 ± 0.5	37 ± 1	Undetectable^(a)^	Undetectable^(a)^	Undetectable^(a)^

^(a)^Undetectable means that the real-time PCR fluorescent signal appears later than that of cut-off assays (see the detection limits and negative control validation)

### Limit of detection

In order to determine the concentration of spiked *E. coli* at which the real-time PCR assay can detect bacterial DNA and the cut-off concentration at which the PCR cannot distinguish spiked bacterial DNA from pre-contaminated microbial DNA, we grew *E. coli* and spiked it in blood samples from healthy donors to establish 20 dilution serials of 1000 CFU/ml, 100 CFU/ml, 10 CFU/ml, 1 CFU/ml and 0 CFU/ml (negative control). These were equally divided into several aliquots to allow independent technical assistants to conduct MCLB-1-based removal of human DNA and DNA extraction. Bacterial DNA was used as template for real-time PCR using primers and probes specific for Enterobacteriaceae and *E. coli*. RT-PCR could detect spiked *E. coli* at concentrations of 100–1000 CFU/ml with a Ct value of 29.2 ± 0.5 at 1000 CFU/ml and of 34.5 ± 0.5 at 100 CFU/ml (Table 2, Additional file 1: Figure S4). In addition, the real-time PCR could still detect 14 out of 20 cases with Ct values of 37 ± 1 at a concentration of 10 CFU/ml. However, the real-time PCR could only detect 7 out of 20 with Ct values of 42 ± 1.3 at a concentration of 1 CFU/ml. These results indicate that our real-time PCR procedure was not able to distinguish samples with spiked bacteria at a concentration of 1 CFU/ml from pre-contaminated bacterial DNA. Therefore, for practical purposes, we set a cut-off of 10 CFU/ml, corresponding to a Ct value of 37 for all pathogens under investigation.

**Table 2:** Detection limits and frequency of detection of spiked *E. coli* dilution series upon in-house human DNA removal step

Spiked *E coli* density (CFU/ml)	1000	100	10	1	0
Average detected Ct	29.2 ± 0.5	34 ± 0.5	37 ± 1	42 ± 1.3	42 ± 1.3
Frequency of detection	20/20	20/20	14/20	7/20	6/20

### Comparison of blood culture versus molecular identification of pathogens

**Fig. 3:**
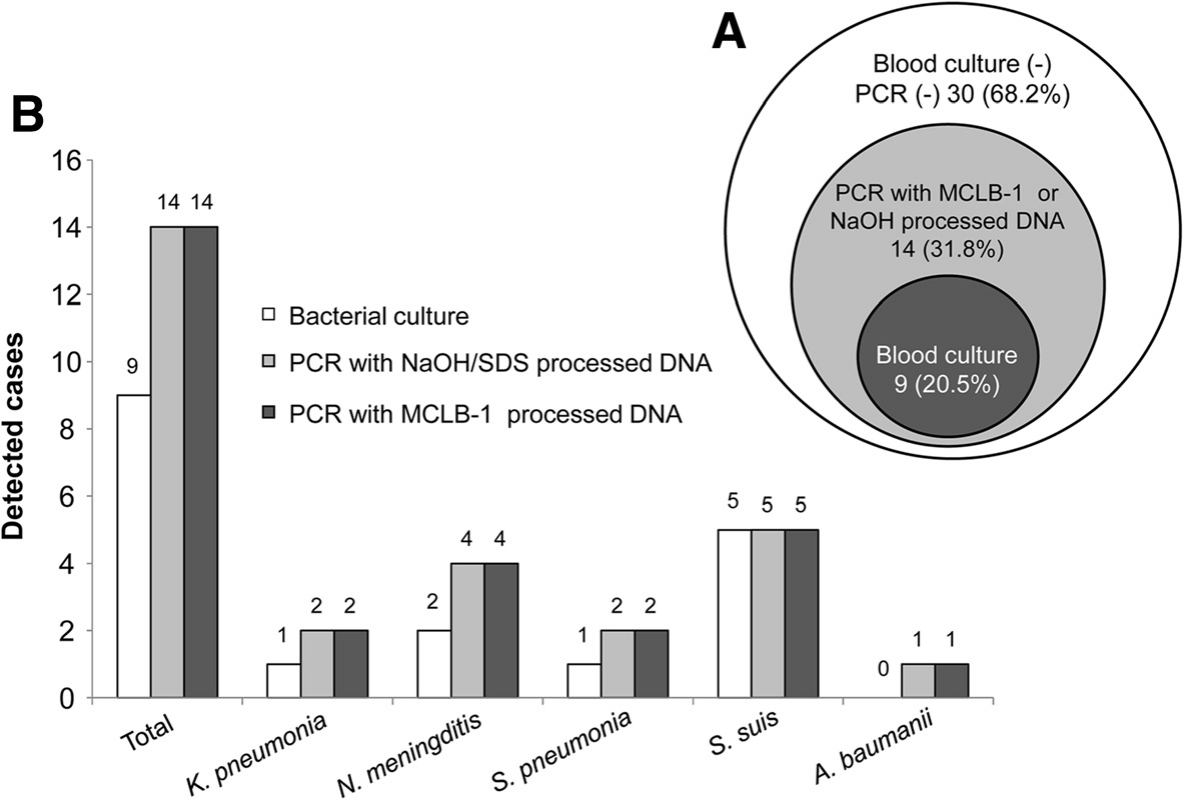
Comparison of diagnostic methodologies for sepsis causing pathogens in CSF samples. **a** Venn diagram shows the diagnostic overlap of the blood culture approach (*dark gray* – arm 1), real-time PCR using conventional NaOH DNA extraction approach (*light gray* – arm 2) and real-time PCR using DNA removed DNA (*light gray* – arm 3). **b** The diagnostic performance are illustrated for classical blood culture (*gray bars*), conventional real-time PCR with total human DNA (*black bar*) and real-time PCR algorithm using MCLB1 treated samples with depleted human DNA

**Fig. 4:**
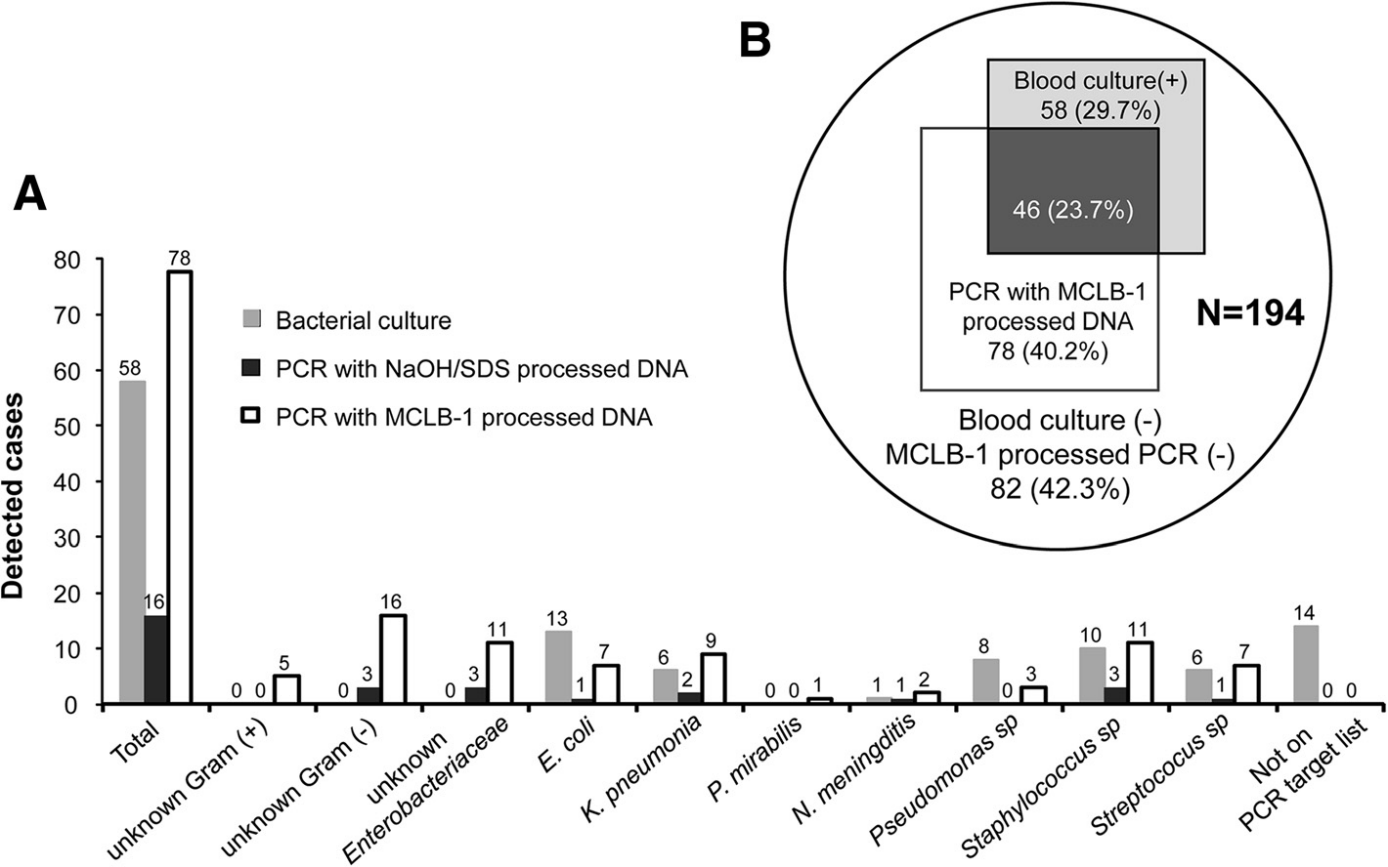
Comparison of diagnostic methodologies for sepsis causative pathogens in human abundance sepsis samples. **a** Venn diagram shows the overlap between PCR using human DNA removed input samples - arm 3 and blood culture method - arm1. **b** The detailed diagnostic performance is illustrated for classical blood culture method (*gray bars*) or conventional real-time PCR approach using DNA extracted from total human blood sepsis samples (*black bar*) or real-time PCR using algorithm using MCLB-1 treated samples

The advantage of human DNA removal prior to PCR diagnosis by treatment with MCLB-1 was validated by using 194 blood samples and 44 CSF samples from septic patients. The samples were divided into three arms, arm 1 for blood culture, arm 2 for direct NaOH/SDS DNA extraction and conventional RT-PCR analysis and arm 3 for MCLB-1 treatment and subsequent RT-PCR detection. Among 44 CSF samples, where human DNA might be present in small amounts only, conventional PCR and the PCR assays with previous MCLB-1 treatment provided identical results (14/44 positive cases). The detection rate by PCR was higher than that obtained by blood culture, which detected bacterial DNA in 9 of the 44 CSF samples only (Fig. 3). However, all positive samples identified by conventional PCR could be confirmed by MCLB-1-processed real-time PCR in the 194 blood samples (Fig. 4). Furthermore, the conventional approach could detect 16 of the 194 samples only, indicating a low power detection rate than that obtained by the MCLB-1-based approach. These results clearly show that efficient removal of human DNA from septicemic blood samples provides a considerable advantage and facilitates PCR diagnoses.

Out of the 194 blood samples obtained from septic patients, 58 (30 %) were positive by blood culture (arm 1), of which 15 (8 %) were positive for other species, namely *Enterobacter sakazaki* (1 case), *L. monocytogenes* (2 cases), *Alcaligenes. faecalis* (1 case), *Pseudomonas. fluorescens* (3 cases), *Stenotrophomonas. maltophilia* (1 case case) and *Candida albicans* (1 case), *Enterobacter. aerogenes* (1 case), *Morganella. morganii* (2 cases), *Burkholderia. pseudomallei* (1 case), *Candida. tropicalis* (1 case) and *Burkholderia. cepacia* (1 case) (Fig. 4a). The in-house real-time PCR methodology using MCLB-1-processed DNA (arm 3) detected 78 (40 %) of 194 BSI samples. The combination of blood culture with MCLB-1-based real-time PCR methods could increase the sensitivity up to 58 % (112 positive cases). We observed that 46 out of 112 positive cases (41 % of positive cases, or 24 % of the total blood samples) were correctly identified between blood culture and MCLB-1 based real-time PCR. Of the 46, 24 (21 % of the positive cases or 12 % of the total blood samples) were identified between the two methods at species level (Fig. 4a, b, Additional file 1: Figure S5).

## Discussion

Our dilution series have shown a strong inhibitory effect of human DNA on subsequent PCR assays targeting bacteria. Once human DNA is removed efficiently from venous blood samples, the detection limit of real-time PCR is enhanced more than ten times. Although removal of human DNA does not improve subsequent PCR detection in samples with low human DNA content such as in CSF, it significantly enhances the diagnostic success of samples with human DNA abundance in septic blood samples.

Bacteremia accounts for a high risk of mortality, both in industrialized and in resource-limited settings [1]. Our genus-specific real-time PCR assays, together with the in-house protocol for removal of human DNA not only provides faster diagnoses, but also increases the sensitivity and specificity in the detection of pathogens compared to culture techniques.

Blood cultures are considered the gold standard for the diagnosis of bacterial or fungal BSI [2]. This method provides results after 24 – 48 h or even a longer period of time, and a tailored therapy can then be adapted based on the presumptive bacterial identification. This method is time consuming with a minimum of 24 to 48 h and treatment options may be compromised severely. Early detection and adequate treatment of BSI are mandatory within the first 6–12 h [20, 21]. The development of rapid diagnostic methods for BSI is important in order to supplement conventional blood culture diagnostics [2]. Nucleic acid-based diagnostic approaches have the potential to address this need [3, 4]. However, the low amount of copies of pathogen DNA and the presence of PCR-inhibitory compounds in blood, including the presence of human DNA, remain challenges [5, 11, 22].

The combination of a mild detergent (MCLB-1, containing 2 M Na_2_CO_2_ pH 9.8, 1 % Triton-X100) applied for 3 min results in removal of approximately 98 % of human DNA from full blood samples. Processing of bacterial DNA prior to molecular detection requires only a short time, which does not cause significant damage of bacterial DNA. In addition, in the presence of a mild detergent, human blood cells are disrupted efficiently to release human DNA. This does not apply to bacterial and fungal cells. An elevated basic pH 9.8 Na_2_CO_3−_ sodium carbonate) ensures successful degradation of human DNA. Thus, intact bacteria and fungi from blood are enriched. Nevertheless, the selective lysis reaction needs to be controlled over time as bacterial cell walls might be lysed upon prolonged exposure.

Current commercial DNA removal kits such as MolYsis Basic (MolZym GmbH & Co. KG, Bremen, Germany) and Looxster (SIRS-lab GmbH, Jena, Germany) work with high blood volumes and require methylated chromatographic columns or DNAses that are patented. Due to significant costs, these kits are commonly not available in low income communities.

Treatment of sepsis usually depends on the presumptive bacterial identification, suggested by Gram staining or/and group specificity of bacteria [23]. Our procedure is a systematic approach, with a stepwise process initiated by real-time PCR reactions to identify Gram-negative, Gram-positive and Enterobacteriacea bacteria. If a patient sample is positive for any group of pathogens, strain-specific Taqman real-time PCR assays are indicated. The diagnostic procedure can be completed within four hours, not only rapidly providing accurate results, but also significantly reducing the cost compared to individual PCR reactions for a large number of pathogens.

In our study and recommended for clinical practice we set a cut-off value of 10 CFU/ml. This limit of detection excludes positive results for patients who are infected with bacterial loads lower than 10 CFU/ml. This explains in part the fact that our molecular method could not recapitulate the positive cases revealed by blood culture. On the other hand, in patients already treated with antibiotics, blood cultures frequently do not lead to reliable diagnoses. This provides an additional explanation for the disagreement between the two methods. Our results are in line with other studies showing that the difference between two diagnostic methodologies fluctuates from 8 % to 80 %, depending on the materials used [11, 24–28]. This explains why the Septifast (Roche Diagnostics) kit is not yet recommended for clinical use in suspected sepsis [28]. Although the overlap between our method and blood cultures was 23.7 % only in our study, the combination of the two methods revealed a diagnostic sensitivity of 57.7 %. This is a significant advantage over with a positive rate of 34 % only and comparability with blood cultures of 8 % [11]. Furthermore, our procedure takes only four hours, which is significantly shorter compared to 18 h required when using any available commercial kits in market [11].

Our detection methodology does not improve the diagnoses of samples with low levels of human DNA. This applies, for example, to CSF specimens. Pretreatment provides an improved DNA template for subsequent PCR and/or real-time PCR, and reaches a sensitivity of 10 CFU/ml.

## Conclusion

We established a simple optimized protocol for human DNA removal from blood sepsis samples. This method provides enriched bacterial DNA for subsequent PCR-based detection of bacterial DNA. With our established protocol, the downstream PCR assays can reach detection limits of 10 CFU/ml and, thus, enhance the positive rate of blood sepsis diagnoses.

## Abbreviations

BSI, Bloodstream infection; CFU, Colony-forming unit; MCLB-1, mammalian cell lysis buffer; NAT, nucleic acid testing; PCR, polymerase chain reaction

## Additional file

Additional file 1: Supplementary study data. (DOC 619 kb)
