# Supplementary material for: Enrichment of bacterial DNA for the diagnosis of blood stream infections
Source: BMC Infect Dis. 2016 May 31;16:235. doi: 10.1186/s12879-016-1568-1 (PMC4888298; doi:10.1186/s12879-016-1568-1)
Supplement: Additional file 1: — Supplementary study data. (DOC 619 kb) [file 12879_2016_1568_MOESM1_ESM.doc]

**Supplementary data**

**Table S1: Optimization of mammalian cell lysis buffer (MCLB)**

| **Solvent name** | **Na_2_CO_3_** | **Triton-X 100 (%)** | **pH** | **Treatment time (mins)** | **Total DNA concentration ug/ul** | **A260/A280** |
| --- | --- | --- | --- | --- | --- | --- |
| MCLB1 | 2000mM | 1 | 9.8 | 3 | Undetectable | 1.3 |
| MCLB2 | 2000mM | 1 | 9.4 | 3 | 20 | 2.7 |
| MCLB3 | 2000mM | 1 | 9 | 3 | 125 | 1.9 |
| MCLB4 | 2000mM | 1 | 10.5 | 3 | 110 | 1.9 |
| MCLB5 | 2000mM | 1 | 11 | 3 | 42 | 1.8 |
| MCLB6 | 2000mM | 1 | 9.8 | 2 | Undetectable | 1.8 |
| MCLB7 | 2000mM | 1 | 9.8 | 3 | Undetectable | 1.8 |
| MCLB8 | 2000mM | 1 | 9.8 | 4 | Undetectable | 1.8 |
| MCLB9 | 2000mM | 1 | 9.8 | 5 | Undetectable | 1.8 |
| MCLB10 | 2000mM | 0.5 | 9.8 | 3 | 165 | 1.8 |
| MCLB11 | 2000mM | 1 | 9.8 | 3 | Undetectable | 1.8 |
| MCLB12 | 2000mM | 1.5 | 9.8 | 3 | Undetectable | 1.8 |
| MCLB13 | 2000mM | 2 | 9.8 | 3 | Undetectable | 2.0 |
| MCLB14 | 1000mM | 1 | 9.8 | 3 | 89 | 2.7 |
| MCLB15 | 1500mM | 1 | 9.8 | 3 | 42 | 2.2 |
| MCLB16 | 2000mM | 1 | 9.8 | 3 | 61 | 2.0 |
| MCLB17 | 2500mM | 1 | 9.8 | 3 | 55 | 2.7 |
| MCLB18 | 3000mM | 1 | 9.8 | 3 | 37 | 2.7 |

**Table S2: Primer and probes used for bacterial ribosome-16S screening and genus specific real-time PCR detection**

| **Bacterial group/ strain** | **Primer/probe 5'-3'** | **Accession Nr./ Target genes** |
| --- | --- | --- |
| **All Gram Negative** | AYGACGTCAAGTCMTCATGG | 16srRNA |
|  | GAGGTGATCCARCCGCA |  |
|  | FAM-TCGTAGTCCGGATYGGABTCTGCA-BHQ1 |  |
| ***Enterobacteriacea*** | GTAAAGYACTTTCAGCGGGGAG | 16srRNA |
|  | ATTCTACCCCCCTCTACRAG |  |
|  | Joe-TGACGTTACCCGCAGAAGAAGCACC-BHQ1 |  |
| *E. coli* | TACGGGAGGCAGCAGT | 16srRNA |
|  | TATTACCGCGGCTGCT |  |
|  | Cy5-AGGGAGTAAAGTTAATACCTTTGCTC-BHQ2 |  |
| *K. pneumoniae* | CCGCGGACTATCTCGACTATAT | AB106869/aldA |
|  | CGATGGCATTATTGGGCGTAAATT |  |
|  | FAM-CGCTGGGCTTAATGACGATGGTATTTCCAGTGAT-BHQ |  |
| *P. mirabilis* | TCACAGTCACCACTAATCTCACGTTGA | Z18752/ureR |
|  | CTGTTGCATAAACAGGGTCTCTTG |  |
|  | FAM-TTTTCCCGACCAAACCGATTGAATTACATACCTTAGT-BHQ |  |
| *Salmollela spp.* | CGACGATTTCTATGCCGCTA | HQ540513/filC |
|  | GTCAAGGTCGCTTGCTAAGT |  |
|  | FAM-CATTGCCACGTGTCAGCTGCACAT-BHQ |  |
| *N. meningitidis* | GCTGCGGTAGGTGGTTCAA | HQ437689/ctrA |
|  | AATGGCTTCAGAAAGCGATAAGCCTCT |  |
|  | FAM-CTGACTCAGGCTTCCCGTAACGCTAAC-BHQ |  |
| *H. influenzae* | TGCGGTAGTGTTAGAAAATGGTATTATG | M19995.1/bexA |
|  | GGACAAACATCACAAGCGGTTA |  |
|  | FAM-ACAAAGCGTATCAATACTACAACGAGACGCAAAAA-BHQ |  |
| *P. aeruginosa* | TACGGGAGGCAGCAGT | 16srRNA |
|  | TATTACCGCGGCTGCT |  |
|  | Joe-GGAAGGGCAGTAAGTTAATACCTTG-BHQ |  |
| *A. baumannii* | TACGGGAGGCAGCAGT | 16srRNA |
|  | TATTACCGCGGCTGCT |  |
|  | FAM-ATACCTAGAGATAGTGGACGTTACTC-BHQ |  |
| **Gram Positive** | GAYGACGTCAARTCMTCATGC | 16srRNA |
|  | GAGGTGATCCARCCGCA |  |
|  | FAM-CGYCKAAGGTGGGAYARATGAT-BHQ1 |  |
| *Staphylococcus sp* | CCGTGTTGAACGTGGTCAAATC | 16srRNA |
|  | GCAACACCACGTAATAAHGCACC |  |
|  | FAM-TGTTGTCACCAGCTTCAGCGTAGTCTAATAATTTACG-BHQ |  |
| *S. aureus* | TACGGGAGGCAGCAGT | 16srRNA |
|  | TATTACCGCGGCTGCT |  |
|  | FAM-GAACATATGTGTAAGTAACTGTGCACA-BHQ |  |
| *S. epidermidis* | TACGGGAGGCAGCAGT | 16srRNA |
|  | TATTACCGCGGCTGCT |  |
|  | FAM-GAACAAATGTGTAAGTAACTATGCACG-BHQ |  |
| *Streptococcus sp* | CAGCWCTTAAAGCTCTTGAAGG | Tuf |
|  | CGGAACATTTCAACACCAGTAAC |  |
|  | FAM-TGGWCGTGGTACWGTWGCTTCAGGACGTAT-BHQ |  |
| *S. pneumoniae* | ACGCAATCTAGCAGATGAAGCA | AM113494/lytA |
|  | TCGTGCGTTTTAATTCCAGCT |  |
|  | FAM-TGCCGAAAACGCTTGATACAGGGAG-BHQ |  |
| *S. suis* | GTGTTCCATGGACAGATAAAGATGG | GU223112/gdh |
|  | AAGACACCTGCATCAAACTGGC |  |
|  | FAM-CCAAGTCAACCGTGGCTACCGTGTTCAGT-BHQ |  |
| *Enterococcus spp.* | TACTTTGTTC AGTTTTGAGA GGT | 16srRNA |
|  | GCAATTGAAC TTATTAAAAA |  |
|  | FAM-CAAACCGAGAACACCGCGTTGAAT-BHQ |  |

**Table S3: Genus specific primers for beta globin and most common sepsis causative pathogens**

| **Bacteria** | **Accession code** | **Forward/Reverse Primer (5' to 3')** | **Size** |
| --- | --- | --- | --- |
| *P. aeruginosa* | AF116258 | CCCGAATGTCGGCATCATTCTC | 411 |
|  |  | CGGTAGACCTCGCGCTTGAA |  |
| *S. aureus* | STAAROA | AAGGGCGAAATAGAAGTGCCGG | 515 |
|  |  | ATGGTCGGTTCCTTAGAAAACAAACTTG |  |
| *A. baumannii* | JX470958 | TTGGGGCCTTTGAGGCTTTAGTG | 599 |
|  |  | TGGTGCAACAAACTCCCATGGT |  |
| *E. coli* | S-uidA | GTCGCGAGTGAAGATCCCTTTC | 773 |
|  |  | GCATTAATGGACTGGATTGGGGC |  |
| *K. pneumoniae* | Kp-aldA | CCTTGTCTTTAAACGCGCGC | 332 |
|  |  | TTTTTCGCCGCAGCGG |  |
| *P. pneumoniae* | LytA | CAACCGTACAGAATGAAGCGGATTAT | 701 |
|  |  | GTCCTTGTACTTGACCCAGCCT |  |
| Human beta globin | MN_000518 | AGAAGAGCCAAGGACAGGTACG | 180 |
|  |  | TGCTAGTGAACACAGTTGTGTCAGA |  |

**Figure S1**: Human DNA residue from pseudo-sepsis samples treated with MCLB-1, MCLB-6, MCLB-7, MCLB-8, MCLB-9, MCLB-11, MCLB-12 and MCLB-13 solvents or total DNA extracted directly by NaOH/SDS protocol was assessed by real-time PCRs using beta globin specific primers.

**Figure S2**: *E. coli* genomic intactness from pseudo-sepsis samples treated with MCLB-1, MCLB-6, MCLB-7, MCLB-8, MCLB-9, MCLB-11, MCLB-12, and MCLB-13 solvents was detected by RT-PCR using *E. coli* specific primers/probe

**Figure S3**: Fluorescent signals from real-time PCRs using DNA prepared by MCLB-1 processed protocol (black bar) or conventional NaOH/SDS protocol ((gray bar) at various density of *E. coli* spiked pseudo-sepsis samples (1000 CFU/ml - left panel), (100 CFU/ml - middle panel), (10 CFU/ml – right panel).

**Figure S4**: Detection limit and negative control validation upon human DNA removal. Left panel fluorescent signals from real-time PCRs using DNA prepared from *E. coli* spiked dilution serial of 1000 CFU/ml, 100CFU/ml, 10CFU/ml, 1 CFU/ml and 0CFU/ml (negative control) by MCLB1 processed protocol from. Right panel fluorescent signals from real-time PCRs using DNA prepared from psedo-sepsis samples spiked with 10CFU/ml, 1 CFU/ml and 0 CFU/ml (negative control) by MCLB-1 processed protocol.

**Figure S5**: Detail agreement between diagnostics result of blood culture vs MCLB-1 based realtime PCR: Samples, which agree at species level are considered full matched, whereas those agree that Gram staining level or group specific level (*Enterobacteriaceae, Staphylococus spp, Pseudomonas spp*) are partial overlap.
